# Polyphyly of *Boehmeria* (Urticaceae) congruent with plastome structural variation

**DOI:** 10.3389/fpls.2024.1297499

**Published:** 2024-07-30

**Authors:** Min Zhan, Ling Xue, Jian-Jun Zhou, Qiang Zhang, Xin-Mei Qin, Xiao-Wen Liao, Lei Wu, Alexander K. Monro, Long-Fei Fu

**Affiliations:** ^1^ College of Forestry, Central South University of Forestry and Technology, Changsha, China; ^2^ Hunan Monitoring Center of Forest Resources and Ecological Environment, Hunan Prospecting Designing and Research General Institute for Agriculture Forestry and Industry, Changsha, China; ^3^ Guangxi Key Laboratory of Plant Conservation and Restoration Ecology in Karst Terrain, Guangxi Institute of Botany, Guangxi Zhuang Autonomous Region and Chinese Academy of Sciences, Guilin, China; ^4^ Royal Botanic Gardens, Kew, Richmond, United Kingdom

**Keywords:** *Boehmeria*, polyphyly, plastome, phylogenetic relationship, structural variations, Urticaceae

## Abstract

*Boehmeria* is a taxonomically challenging group within the nettle family (Urticaceae). The polyphyly of the genus has been proposed by previous studies with respect to five genera (*Debregeasia*, *Cypholophus*, *Sarcochlamys*, *Archiboehmeria*, and *Astrothalamus*). Extensive homoplasy of morphological characters has made generic delimitation problematic. Previous studies in other plant groups suggest that plastome structural variations have the potential to provide characters useful in reconstructing evolutionary relationships. We aimed to test this across *Boehmeria* and its allied genera by mapping plastome structural variations onto a resolved strongly supported phylogeny. In doing so, we expanded the sampling of the plastome to include *Cypholophus*, *Sarcochlamys*, *Archiboehmeria*, and *Astrothalamus* for the first time. The results of our phylogenomic analyses provide strong support for *Sarcochlamys* as being more closely related to *Leucosyke puya* than to *Boehmeria* and for the clustering of *Boehmeria* s.l. into four subclades. The sizes of the plastomes in *Boehmeria* s.l. ranged from 142,627 bp to 170,958 bp. The plastomes recovered a typical quadripartite structure comprising 127~146 genes. We observe several obvious structural variations across the taxa such as gene loss and multiple gene duplication, inverted repeat (IR) contraction and wide expansions, and inversions. Moreover, we recover a trend for these variations that the early clades were relatively conserved in evolution, whereas the later diverging clades were variable. We propose that the structural variations documented may be linked to the adaptation of *Boehmeria* s.l. to a wide range of habitats, from moist broadleaf forests in Asia to xeric shrublands and deserts in Africa. This study confirms that variation in plastome gene loss/duplication, IR contraction/expansion, and inversions can provide evidence useful for the reconstruction of evolutionary relationships.

## 1 Introduction

*Boehmeria* Jacq., the largest genus in the tribe Boehmerieae (Urticaceae) with 51 accepted species (POWO, 2024), is native to Asia, Africa, and the Americas. It encompasses a wide range of life forms including perennial herbs, shrubs, and trees. *Boehmeria* occurs in diverse biomes, tropical and subtropical moist broadleaf forests, temperate broadleaf forests, tropical dry broadleaf forests, and subtropical dry broadleaf forests (Kravtsova et al., 2000; Wilmot-Dear and Friis, 2013). *Boehmeria* species have been utilized for a multitude of purposes such as fiber production, forage, restoration, and consumption as a green vegetable and beverage, as well as in traditional medicine (Rehman et al., 2019; Arsul et al., 2021; Lee et al., 2022; Liu et al., 2022; Zhu et al., 2022). Notably, two species, *Boehmeria oblongifolia* and *B. leiophylla*, are classified as second-class protected plants in China (https://www.gov.cn/zhengce/zhengceku/2021–09/09/content_5636409.htm).

**Figure 1:**
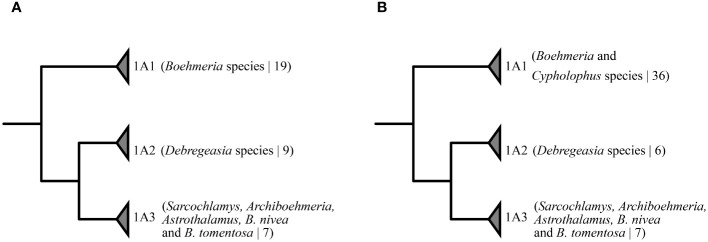
Phylogenetic relationships of the three clades formed by *Boehmeria* and its related genera from previous analyses based on combined data of four chloroplast, two nuclear, and one mitochondrial loci: **(A)** Wu et al. (2013), (2015); **(B)** Wu et al. (2018b). Numeric values in brackets correspond to the total number of accessions sampled in the clade.

*Boehmeria* is considered a taxonomically challenging group, with uncertainty over the rank of taxonomic entities and a non-phylogenetic infrageneric classification, and as such warrants further research (Kravtsova et al., 2000; Wilmot-Dear et al., 2010; Wilmot-Dear and Friis, 2013). Recent phylogenetic studies based on several loci have revealed the poly- or paraphyletic nature of the genus in relation to five other genera (*Debregeasia*, *Cypholophus*, *Sarcochlamys*, *Archiboehmeria*, and *Astrothalamus*) (Wu et al., 2013, 2015, 2018b). In these studies, *Boehmeria* and allied genera are consistently recovered in three strongly supported clades (see **Figures 1A, B**: 1A1 to 1A3) in which clade 1A1 was inferred as a sister group to a group comprising clades 1A2 and 1A3. Wu et al. (2015) evaluated the informativeness of 19 morphological characters, all of which were recovered as homoplastic. *Cypholophus* (approximately 30 species) and *Debregeasia* (nine species) are the only genera with a higher species diversity, while the remaining three genera are monotypic (POWO, 2024). Of the genera allied to *Boehmeria*, all are shrubs or trees, primarily distributed in the tropical and subtropical moist broadleaf forests of Indomalaya and the Palearctic, and only *Debregeasia* extends into dry biomes (dry broadleaf forests, xeric shrublands, and deserts in the Afrotropics) (POWO, 2024).

Plastomes have emerged as valuable tools in resolving phylogenetic relationships in plants. Their moderate nucleotide substitution rates, smaller genome sizes, and high copy number make them ideal for phylogenetic study (Twyford and Ness, 2017; Guo et al., 2022). While plastome structural variations have been identified as phylogenetically informative characters that can aid in understanding evolutionary relationships (Jansen and Palmer, 1987; Wu et al., 2024), they have been rarely used to do so. Plastome structural variations include, among others, contraction or expansion of inverted repeats (IRs), gene loss or duplication, and inversions (Villarreal et al., 2013; Dugas et al., 2015; Choi and Choi, 2017; Sinn et al., 2018; Wu et al., 2024). Plastome evolution has been linked to speciation events, suggesting its role in diversification within plant lineages (Bock et al., 2014; Scobeyeva et al., 2021). Although a few *Boehmeria* plastomes have been assembled, analyses have focused on single species and/or inferred phylogenetic relationships based on very limited taxon sampling (Wu et al., 2018a; He et al., 2021). Whole plastome data have been used to elucidate phylogenetic relationships within the Urticaceae, specifically the Urticeae tribe and three specific genera (*Oreocnide*, *Debregeasia*, and *Pilea*) (Wang et al., 2020; Li et al., 2021; Ogoma et al., 2022; Wu et al., 2022). Little attention, however, has been given to the plastome’s structural variations in these studies, with only IR expansion/contraction, a few gene losses, and inversions being mentioned. In summary, while plastome data have been utilized for phylogenetic inference within the Urticaceae family, including *Boehmeria*, there remains an untested opportunity for the application of plastome structural information.

For the above reasons, we sampled species of *Boehmeria* and the related genera (*Debregeasia*, *Cypholophus*, *Archiboehmeria*, *Astrothalamus*, and *Sarcochlamys*), aiming to evaluate the power of the plastome dataset in elucidating evolutionary relationships. Our main tasks were as follows: 1) reconstruct robust phylogenetic relationships with different data matrixes and analytical methods and 2) investigate plastome structural variations.

## 2 Materials and methods

### 2.1 Sampling and sequencing

Previous studies have recovered a polyphyletic *Boehmeria* related to five other genera. We re-evaluated these relationships using whole plastome data. A plastome phylogenomic study about one of these genera (*Debregeasia*) has been conducted previously (Wang et al., 2020). Thus, we sampled representatives from the remaining genera, and a total of 11 newly sequenced species were included (six from *Boehmeria* and the remaining five from *Debregeasia*, *Cypholophus*, *Archiboehmeria*, *Astrothalamus*, and *Sarcochlamys*, respectively). Detailed sampling information is provided in **Supplementary Table 1**. Total genomic DNA (gDNA) was extracted from silica-gel-dried leaves using the modified CTAB method (Arseneau et al., 2017). The quality and quantity of DNA were measured on 1% Tris–acetate–ethylenediamine tetraacetic acid (TAE) agarose gels using the Qubit fluorometric quantification (Invitrogen, Carlsbad, CA, United States). The gDNA was fragmented and library size was selected for 350-bp inserts. Sequencing with 2 × 150-bp paired-end (PE) reads was performed on the Illumina HiSeq 2500/X-Ten at the Beijing Genomics Institute (BGI) in Shenzhen, China. The raw data underwent preprocessing to eliminate adapter sequences and low-quality bases and reads, yielding approximately 2 Gb of clean data per sample.

### 2.2 Assembly and annotation

Clean reads were used to conduct *de-novo* assembly in GetOrganelle v1.7.6.1 with a kmer length of 65–121 bp (Jin et al., 2020). Next, the well-assembled circular sequence was annotated using both PGA and CPGAVAS2 with *Boehmeria spicata* (NC_036989), *B. umbrosa* (NC_036990), and *Debregeasia saeneb* (NC_062311) as reference genomes (Qu et al., 2019; Shi et al., 2019). Subsequently, we examined the accuracy and did manual corrections, where necessary, in CPGView and Geneious v9.0.2 (Kearse et al., 2012; Liu et al., 2023). The annotated plastomes were submitted to NCBI to acquire accession numbers (**Supplementary Table 2**).

### 2.3 Phylogenetic inference based on plastome data

#### 2.3.1 Other published plastome data sampling and reannotation

To clarify and confirm the relationships of *Boehmeria* and other genera, 22 representatives from other genera and tribes in Urticaceae as well as eight and six published *Boehmeria* and *Debregeasia* species, respectively, were included as ingroups, and three species from Moraceae were used as outgroups. Accession numbers can be found in **Supplementary Table 2**.

Although plastomes are relatively conservative, structural variations (like inversion and IR contraction/expansion) are very common in some taxa, which will lead to aligning difficulty and errors when applying whole plastomes to infer phylogenetic relationships. Considering this, we usually separately extract and align protein-coding sequences (CDS), genes, and intergenetic regions (IGS) according to the annotation information of plastomes. However, annotation errors frequently occur in published plastomes, which is not conducive to downstream analysis. Therefore, we checked and re-annotated these downloaded plastomes in Geneious following the steps in Qu et al. (2023).

#### 2.3.2 Phylogeny reconstruction

Previous studies have shown changed substitution rates for genes transferred to the IR or SC region due to the boundary shift (Guo et al., 2021), indicating that these genes in different taxa or lineage may suffer from heterotachy (Wang et al., 2019; Kapli et al., 2020; Steenwyk et al., 2023). Applying such genes can impact the phylogenetic reconstruction (Lockhart et al., 2006; Zhong et al., 2010; Wu et al., 2011). Therefore, we prepared two matrixes to test whether boundary genes can twist phylogenetic relationships in our studied taxa—matrix1: 78-CDS (common CDS) and matrix2: 59-CDS (removing CDS from 19 genes located at IR/SC boundaries). To fully utilize phylogenetic information from the whole plastome, another two matrixes were also prepared—matrix3: genes+IGS and matrix4: genes+IGS-SNP.

Empirical studies on the structural and functional attributes of plastomes indicate that the plastome may not evolve as a single locus and may be subject to divergent evolutionary pressures (Sloan et al., 2012; Dugas et al., 2015; Weng et al., 2016; Gonçalves et al., 2019). Additionally, plastid gene tree discordance has been found in several studies (Lockhart et al., 2006; Wu et al., 2011; Zhang et al., 2020). Therefore, we employed both concatenation and coalescent-based methods. Sequence alignment was carried out in MAFFT v7.407 (Katoh and Standley, 2013), and poorly aligned regions were trimmed with default settings in trimAL v1.4.1 (Capella-Gutiérrez et al., 2009). For concatenation-based methods, Geneious Prime v9.0.2 was used to concatenate data into one matrix (Kearse et al., 2012). The maximum likelihood (ML) tree was constructed in IQ-TREE v1.6.12 (Nguyen et al., 2015) with 5,000 bootstrap replicates and automatically selecting the best model. The BI tree was obtained in MrBayes v3.2.7a (Ronquist et al., 2012) by running 100 million generations and sampling every 1,000 generations. The best substitution model was determined based on the Bayesian information criterion (BIC) (Aho et al., 2014) in jModelTest2 v. 2.1.7 (Darriba et al., 2012). All best-fit models can be found in **Table 1**. Two independent runs were performed, each consisting of four Markov chain Monte Carlo (MCMC) chains. The beginning 25% of trees were discarded as burn-in, while the remaining trees were used for generating a consensus tree. The convergence of the MCMC chains of each run was determined when the average standard deviation of split frequencies (ASDSF) achieved ≤0.01. For coalescent-based methods, single gene trees were inferred in IQ-TREE with 1,000 bootstrap replicates and automatically selecting the best models. Nodes with less than 20% support were collapsed using Newick Utilities v1.6 (Junier and Zdobnov, 2010) as this can help improve gene tree accuracy. Species tree inference was conducted in ASTRAL III v5.7.8 (Zhang et al., 2018), node support was assessed by local posterior probability (LPP; Sayyari and Mirarab, 2016), and normalized quartet score (NQS) and quartet frequencies were used to reflect the level of gene tree discordance.

**Table 1:** NQSs and best substitution models were selected for four data matrixes.

Dataset	Coalescent method	Concatenated method	
	Normalized quartet score	Best substitution model for the ML tree	Best substitution model for the BI tree
Matrix1	0.935	GTR+F+I+R3	GTR+I+G
Matrix2	0.925	GTR+F+I+R4	GTR+I+G
Matrix3	0.942	GTR+F+I+R4	GTR+I+G
Matrix4	0.934	K3Pu+F+ASC+R3	GTR+I+G

### 2.4 Plastome structural variation analyses

According to the delimitation results of plastome-based phylogenetic inference, a total of 23 plastomes were used to perform structural variation analyses. Basic structural features were summarized in Geneious, including plastome size, GC content (of the whole, SC, and IR), gene numbers, and gene duplication and loss. The four junctions between IR and SC of those 23 plastomes were compared and visualized in CPJSDRAW (Li et al., 2023). Whole genome alignments for the 23 plastomes and the reference *Hemistylus odontophylla* (MN189963) were performed using progressiveMauve v. 2.3.1 (Darling et al., 2004).

## 3 Results

### 3.1 Assembly and annotation condition

Four genera (*Cypholophus* and three monotypic genera: *Archiboehmeria*, *Astrothalamus*, and *Sarcochlamys*) are sequenced at the genomic level for the first time. All samples were successfully assembled except *Astrothalamus reticulatus*, with only a few scaffolds assembled. These successfully assembled plastomes have a typical quadripartite structure ranging from 142,627 bp to 170,051 bp, containing 82–98 protein-coding genes (PCGs), 37 tRNAs, and 8 rRNAs (**Table 2**). Although *A. reticulatus* is not completely assembled, 48 PCGs are retained from scaffolds, which still can be used in the downstream analysis.

**Table 2:** Comparison of basic plastome features within the four clades.

Clades	Species	Whole size	LSC length	SSC length	IR length	GC%	LSC GC%	SSC GC%	IR GC%	Number of genes	Protein-coding genes	tRNAs	rRNAs
B1	Boehmeria zollingeriana*	154,923	84,501	18,658	25,882	36.02	33.63	29	42.46	129	84	37	8
	Boehmeria glomerulifera*	155,320	84,859	18,661	25,900	36	33.59	29.01	42.47	129	84	37	8
C	Cypholophus macrocephalus*	142,627	89,709	19,022	16,948	35.58	33.51	28.68	44.94	127	82	37	8
B2	*Boehmeria spicata*	170,958	70,994	18,478	40,743	35.32	33.1	28.31	38.84	145	100	37	8
	Boehmeria macrophylla*	161,166	82,512	18,468	30,093	35.57	33.51	28.57	40.54	134	89	37	8
	Boehmeria clidemioides*	170,051	72,707	18,462	39,441	35.47	33.35	28.51	39.05	143	98	37	8
	*Boehmeria umbrosa*	170,920	68,844	18,462	41,807	35.53	33.45	28.59	38.78	146	101	37	8
	*Boehmeria dolichostachya*	161,904	80,148	18,404	31,676	35.68	33.46	28.59	40.54	135	89	38	8
	Boehmeria densiflora*	159,231	81,082	18,315	29,917	35.35	33.28	28.16	40.35	133	88	37	8
	*Boehmeria verticillata* sp. nov.*	161,973	80,186	18,055	31,866	35.55	33.39	28.69	40.21	136	91	37	8
	*Boehmeria japonica*	156,266	83,158	18,392	27,358	35.66	33.34	28.55	41.58	132	87	37	8
B3-D	Archiboehmeria atrata*	156,030	85,710	18,924	25,698	36.33	34.04	29.63	42.63	129	84	37	8
	*Boehmeria nivea*	155,959	85,639	18,924	25,698	36.34	34.05	29.62	42.63	129	84	37	8
	Boehmeria nivea *var.* tenacissima	155,807	85,721	18,690	25,698	36.36	34.03	29.77	42.62	129	84	37	8
	*Boehmeria tomentosa*	154,938	85,720	17,822	25,698	36.41	34.03	29.95	42.63	128	84	36	8
	Boehmeria nivea *var.* nipononivea	155,806	85,717	18,693	25,698	36.36	34.03	29.78	42.63	129	84	37	8
	*Debregeasia saeneb*	155,780	85,499	18,983	25,649	36.29	34	29.45	42.64	129	84	37	8
	*Debregeasia longifolia*	155,904	85,627	18,979	25,649	36.28	33.98	29.41	42.65	129	84	37	8
	*Debregeasia squamata*	156,065	85,649	19,088	25,664	36.28	33.99	29.41	42.65	129	84	37	8
	*Debregeasia* sp.*	156,013	85,601	19,084	25,664	36.28	34	29.4	42.65	129	84	37	8
	*Debregeasia elliptica*	155,940	85,538	19,074	25,664	36.29	34.01	29.42	42.65	129	84	37	8
	*Debregeasia hekouensis*	155,941	85,528	19,085	25,664	36.29	34.01	29.4	42.65	129	84	37	8
	*Debregeasia orientalis*	160,283	88,506	20,049	25,864	36.13	33.88	29.04	42.74	129	84	37	8

*Asterisks indicate the newly sequenced species.

### 3.2 Phylogenetic relationships

**Figure 2:**
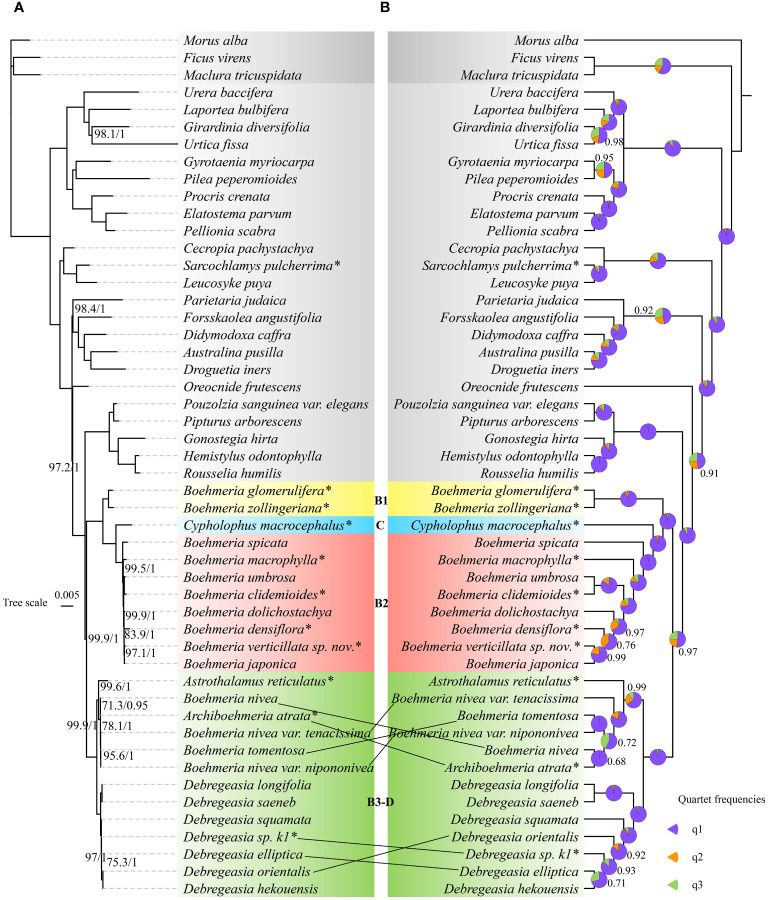
Phylogenetic relationships of *Boehmeria* and its related genera inferred by matrix1 (78-CDS): **(A)** concatenated tree produced by maximum likelihood (ML) analysis—numbers associated with branches (ML_BS/BI_PP) are assessed by maximum likelihood bootstrap (ML_BS) and Bayesian posterior probabilities (BI_PP); **(B)** coalescent tree—numbers associated with branches denote local posterior probability (LPP) support values, and pie charts show the relative frequencies of the three quartet topologies around the branch (purple = congruent with the species tree, orange = first alternative topology, green = second alternative topology). Branches with no support values are maximally supported. *Asterisks indicate the newly sequenced species.

Both matrix1 (**Figures 2A, B**) and matrix2 (**Supplementary Figures 1A, B**) recovered congruent tree topologies in concatenated and coalescent methods, respectively, with most nodes receiving high support values (MLBS = 100; PP = 1; LPP = 1). An exception was the relationship between *Archiboehmeria atrata* and *B. nivea*: in the concatenated ML tree of matrix2, the two were separated with no support value while they clustered into sisters in all other trees. The overall support value of matrix2 was moderately lower than matrix1.

Matrix3 (**Supplementary Figures 2A, B**) and matrix4 (**Supplementary Figures 3A, B**) present the same topologies as matrix1 in concatenated methods with only a few moderate differences in support values. In the coalescent method, these three matrixes also show congruent topologies. All NQSs of the coalescent method are >0.92 (**Table 1**). The topologies of concatenated and coalescent methods are also congruent.

All trees recovered *Boehmeria* as polyphyletic with respect to *Astrothalamus*, *Archiboehmeria*, *Cypholophus*, and *Debregeasia*, while *Sarcochlamys pulcherrima* was inferred as sister to *Leucosyke puya* (non-Boehmeriaea species) with full support (MLBS = 100; PP = 1; LPP = 1). *Boehmeria* and its related genera (*Boehmeria* sensu lato) formed two large clades. To better aid subsequent analyses, we further divided them into four subclades, namely, B1, C, B2, and B3-D (**Figure 2**). Clade B1 consisted of two *Boehmeria* species, clade C contained *Cypholophus* species, and clade B2 consisted of eight *Boehmeria* species; clade B1 and clade C were successive sisters to clade B2. Clade B3-D formed two subclades (B3 and D), one containing *Archiboehmeria*, *Astrothalamus*, *Boehmeria tomentosa*, and *B. nivea* and the other containing exclusively *Debregeasia* species. Clade B3-D was a sister group to the clade consisting of clades B1, C, and B2.

### 3.3 Plastome structural variations

#### 3.3.1 General plastome characteristics of *Boehmeria* s.l.

**Figure 3:**
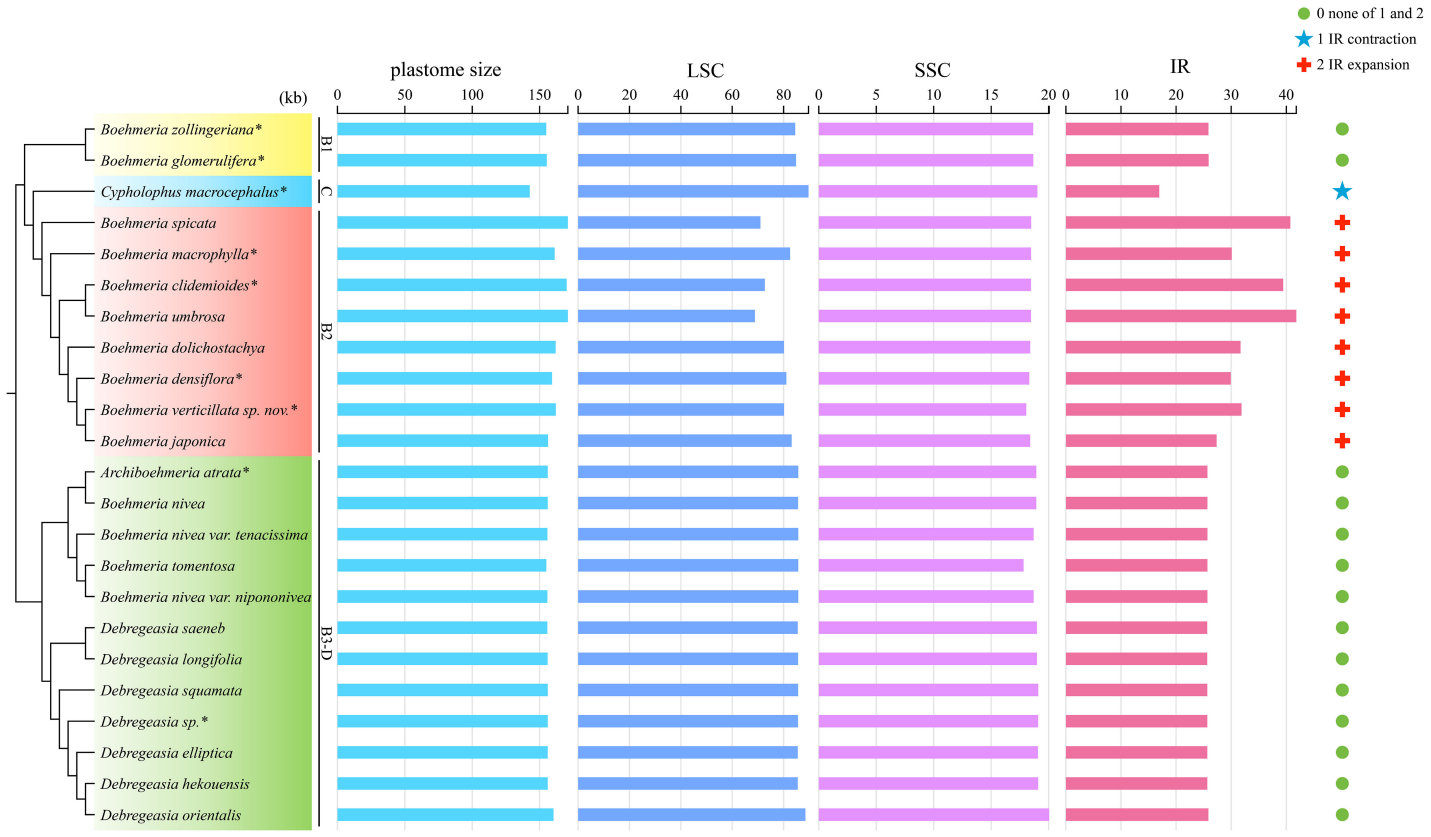
Plastome size variation and IR contraction and expansion in the four clades. The cladogram (left) was converted from the concatenated method phylogram (**Figure 2A**); the histogram (medium) shows the comparison of the whole size and three different regions of plastomes; the symbol matrix (right) shows the conditions of IR contraction and expansion. kb, kilobases. *Asterisks indicate the newly sequenced species.

Great variations in the plastome size were found in *Boehmeria* s.l. (**Table 2**). The smallest plastome occurred in clade C (*Cypholophus macrocephalus*, 142,627 bp), and the largest occurred in clade B2 (*B. spicata*, 170,958 bp), which showed larger plastome size than the other three clades. The smallest IR occurred also in clade C (*C. macrocephalus*, 16,948 bp), and the largest in clade B2 (*B. umbrosa*, 41,807 bp), which showed a larger IR size than the other three clades (**Table 2**, **Figure 3**). The LSC and SSC regions showed relatively little variation, ranging from 68,844 bp to 88,506 bp and 17,822 bp to 20,049 bp, respectively. The GC content of plastomes also varied among these clades, with clade B2 having a lower GC content in the whole plastome, LSC, SSC, and IR regions compared to the other clades (**Table 2**).

#### 3.3.2 Multiple gene duplications and several gene losses

**Figure 4:**
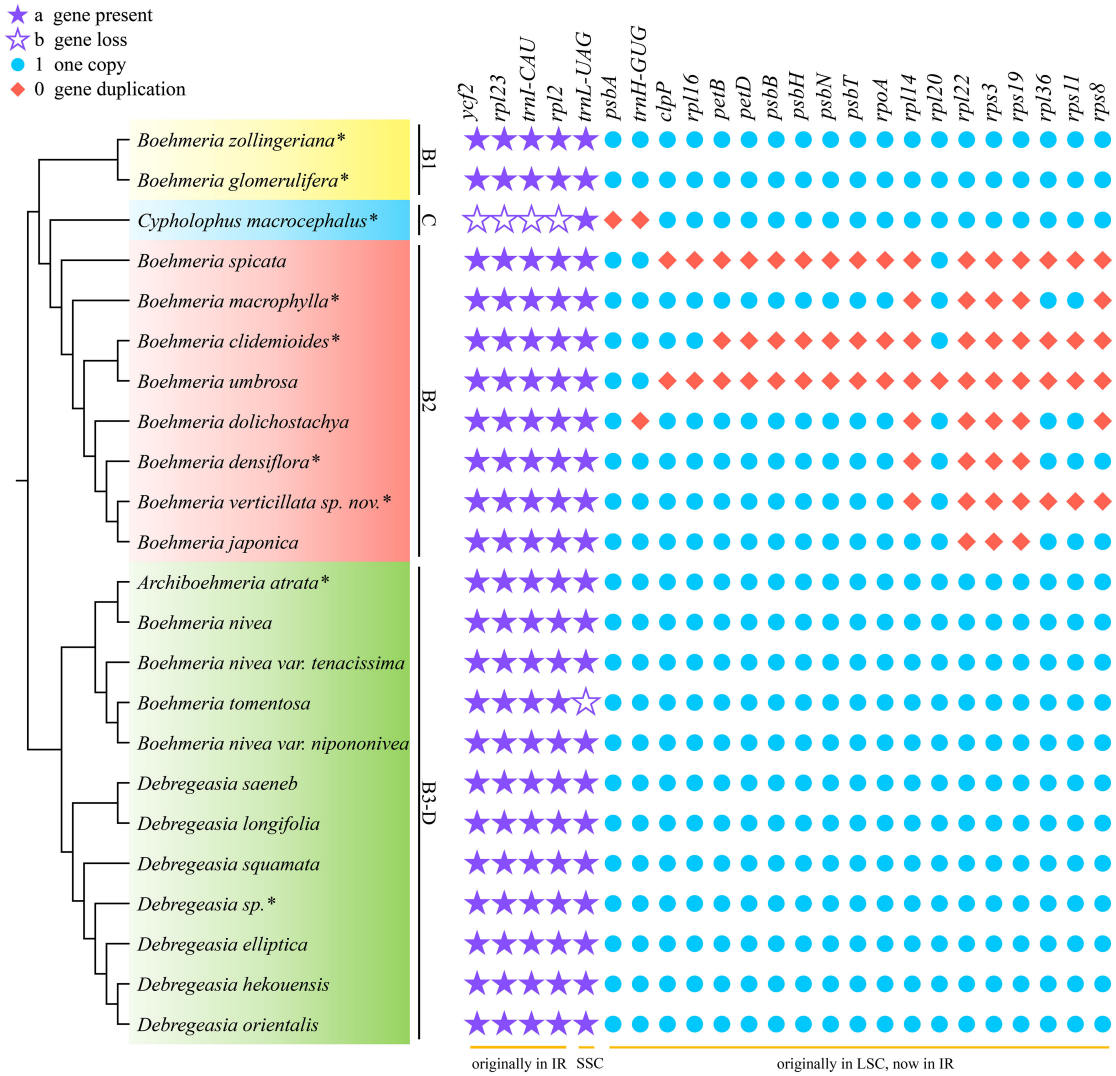
Gene loss and duplication in the four clades. The cladogram (left) was converted from the concatenated method phylogram (**Figure 2A**); the symbol matrix (right) shows the conditions of gene loss and duplication. *Asterisks indicate the newly sequenced species.

Comparison of the 23 plastomes of *Boehmeria* s.l. showed that they encoded a set of 127 to 146 genes, including 84 to 101 PCGs, 36 to 38 tRNAs, and 8 rRNAs (**Table 2**). Clades B1 and B3-D had the same conserved 84 PCGs, while clades C and B2 experienced PCG loss and duplication (**Table 2**, **Figure 4**). Clade C experienced both gene loss and duplication, including loss of one copy of *ycf2*, *rpl2*, *rpl23*, and *trnI-CAU* and duplication of *psbA* and *trnH-GUG*. Clade B2 experienced multiple gene duplications, including a total of 17 PCGs and 1 tRNA (**Figure 4**). These gene losses and duplications were all caused by IR boundary shifts. The LSC/IRb (JLB) border of *C. macrocephalus* (clade C) moved toward IRb leading to four genes (*ycf2*, *rpl23*, *rpl2*, and *trnI-CAU*), originally located in IRb, being relocated in the LSC region; therefore, each lost one copy in the IRa. The IRa/LSC (JLA) border moved toward LSC leading to two genes (*psbA*, *trnH-GUG*), originally located in the LSC, being relocated in IRa; thus, each had the second copy in the IRb. The multiple gene duplications of all members in clade B2 were only due to the JLB border moving toward LSC resulting in multiple genes originally located in the LSC being relocated in IRb, and thus, each had an extra copy in IRa. Additionally, *B. tomentosa* of clade B3-D lost *trnL-UAG*, which was the only observed loss in clade B3-D.

#### 3.3.3 IR expansion/contraction and different boundary types

Both the expansion and contraction of IR were recovered in our studied taxa. Clades B1 and B3-D exhibited relatively conserved IR sizes with averages of 25,891 bp and 25,692 bp, respectively. The IR of *C. macrocephalus* in clade C contracted to 16,948 bp, while its sister clade (B2) showed a marked expansion with an average IR size of 34,113 bp (**Table 2**, **Figure 3**).

**Figure 5:**
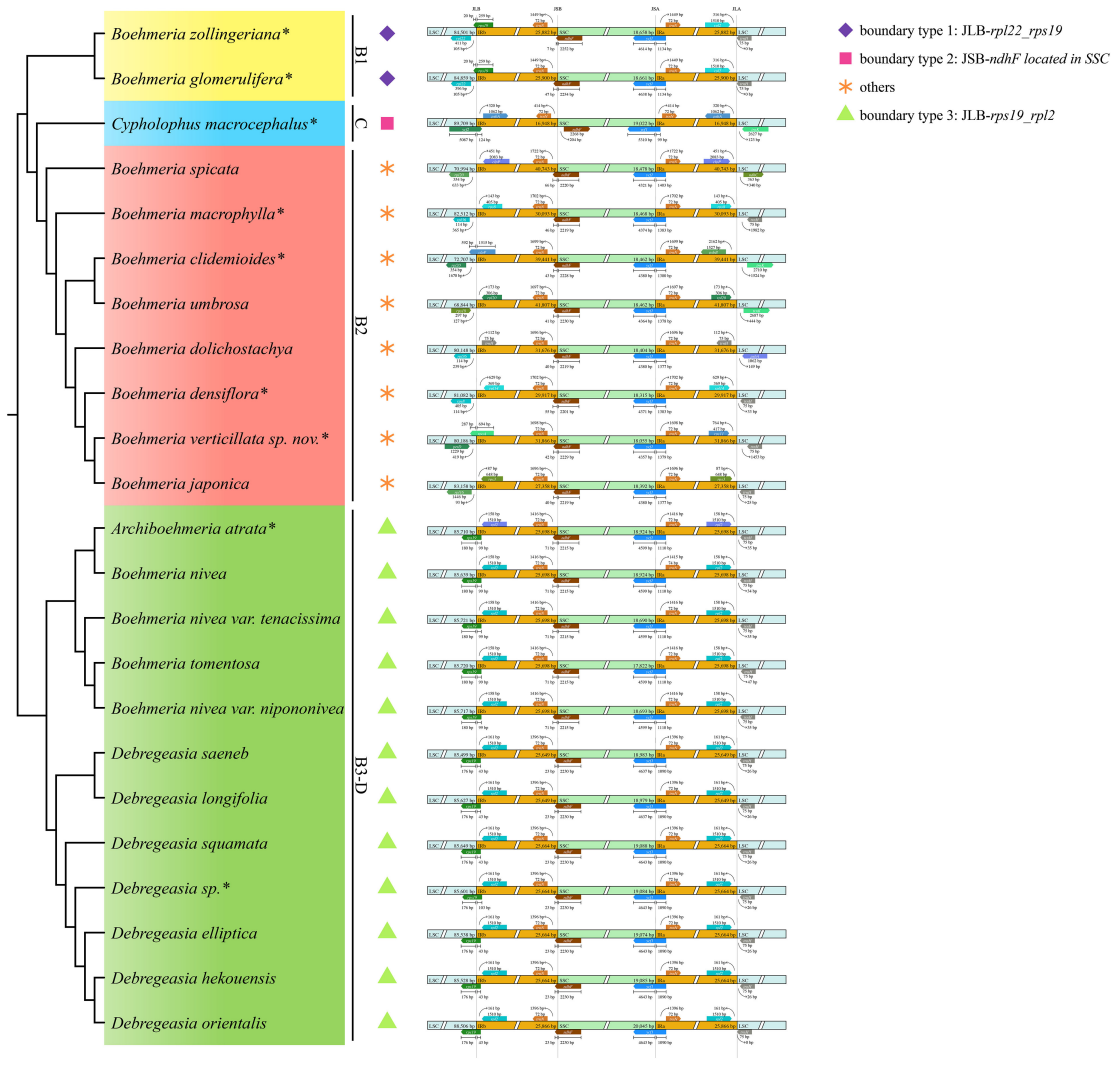
IR/SC boundary comparison in the four clades. The cladogram (left) was converted from the concatenated method phylogram (**Figure 2A**); the symbol matrix (medium) shows the different IR/SC boundary types; the image (right) shows the four junctions of plastomes; JLB, JSB, JSA, and JLA represent the junction sites of LSC/IRb, IRb/SSC, SSC/IRa, and IRa/LSC, respectively. *Asterisks indicate the newly sequenced species.

The SSC/IRa (JSA) boundary was conservative in all four clades with *ycf1* located here (**Figure 5**). IRb/SSC (JSB) was also conservative with *ndhF* across the border, except for *C. macrocephalus* of clade C with this gene completely located in the SSC region; the direction of it was forward (boundary type 2) (**Figure 5**). JLA was conservative in clades B1 and B3-D with *rpl2* and *trnH-GUG* near this border, but it was diverse in clades C and B2. JLB was also conservative in clades B1 and B3-D, with *rpl22* near and *rps19* across the boundary (boundary type 1) and *rps19* across and *rpl2* near the boundary (boundary type 3), but JLB was also diverse in clades C and B2.

#### 3.3.4 Single gene inversions and large-scale inversions

**Figure 6:**
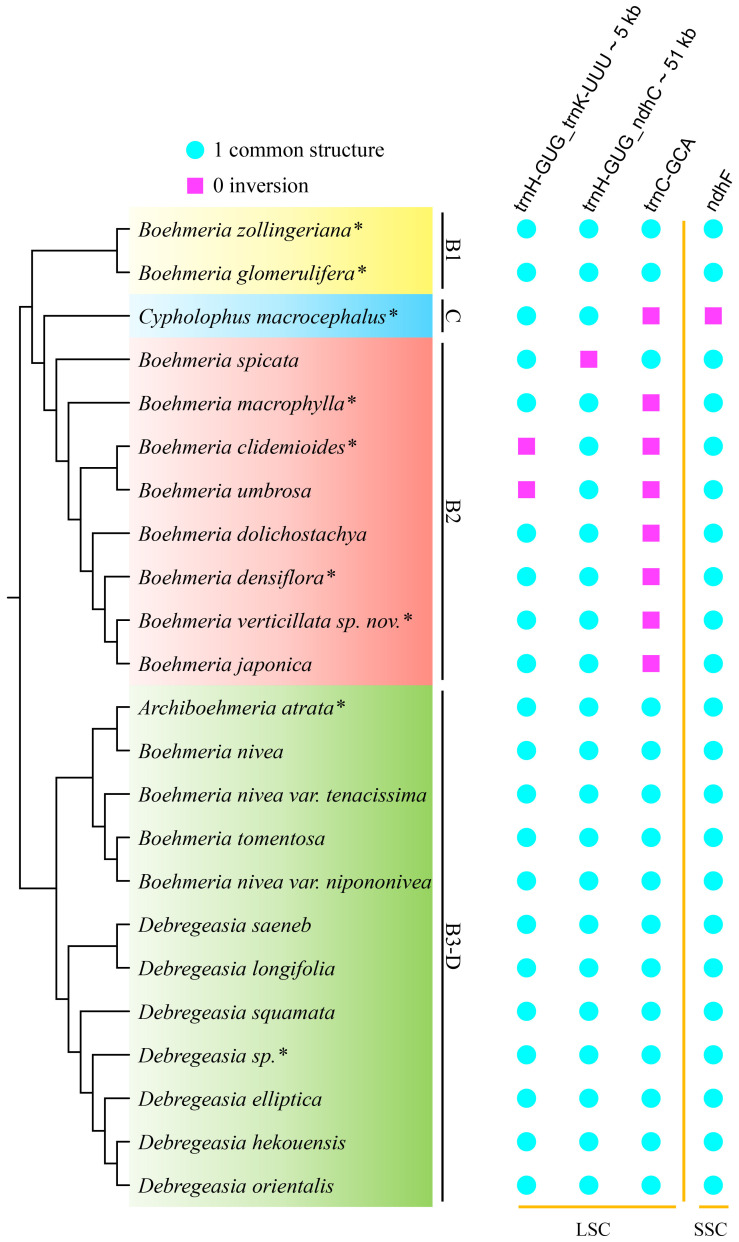
Inversions in the four clades. The cladogram (left) was converted from the concatenated method phylogram (**Figure 2A**); the symbol matrix (right) shows the conditions of inversions. *Asterisks indicate the newly sequenced species.

Clades B1 and B3-D were conservative with no inversion, while clade C and all members of clade B2 showed inversions (**Figure 6**). A total of four inversion events were detected, including single gene inversions (*ndhF* and *trnC-GCA*) and large-scale inversions (**Figure 6**; **Supplementary Figure 4**). The *ndhF* inversion solely occurred in *C. macrocephalus*; the *trnC-GCA* inversion was shared by clades C and B2. *Boehmeria spicata* of clade B2 possessed two inversions (*trnC-GCA* inversion; the large-scale inversion *trnH-GUG_ndhC*, which overlapped with the former inversion), causing the direction of *trnC-GCA* being finally different from other members in this clade. The large-scale inversion of *trnH-GUG_ndhC* (~51 kb) was recovered in *B. spicata*, while the remaining large-scale inversion of *trnH-GUG_trnK-UUU* (~5 kb) was shared by *B. clidemioides* and *B. umbrosa*. All these inversions occurred in the LSC region.

## 4 Discussion

### 4.1 Plastome phylogenomics inferred the polyphyly of *Boehmeria*

Our results showed congruent topologies between matrix1 and matrix2 both in the concatenated and coalescent methods, but the support values become lower after removing boundary genes. This probably means that the boundary genes may not suffer from heterotachy or the effect of heterotachy was too little to twist phylogenetic reconstruction in our studied taxa. Moreover, the NQSs are all >0.92, indicating only slight gene incongruence. Still, the use of such genes needs to be done cautiously, since they can lead to incongruent and misleading tree topologies (Lockhart et al., 2006; Wu et al., 2011).

Our four data matrixes derived from the plastome recovered consistent tree topologies in both concatenated and coalescent methods with strong support at most nodes, revealing *Boehmeria* as polyphyletic with respect to the other four genera (*Cypholophus*, *Astrothalamus*, *Archiboehmeria*, and *Debregeasia*). Species of *Boehmeria* and the four genera formed two large clades: one is B1-C-B2, which is dominated by *Boehmeria* species and corresponds to clade 1A1 in Wu et al. (2013), (2015), (2018b); the other formed B3 and D, which corresponds to 1A3 and 1A2 of Wu et al. (2013), (2015), (2018b). *Cypholophus* (C) was nested within *Boehmeria*, which was consistent with Wu et al. (2018b). *Archiboehmeria* was recovered within a clade that also includes *B. nivea* and *Astrothalamus*, and this clade (B3) was recovered as a sister to *Debregeasia* (D). As above, this was consistent with Wu et al. (2013), (2015), (2018b). The position of *Sarcochlamys*, sister to *Leucosyke* within a clade sister to the Cecropieae, differed from Wu et al. (2013), (2015), (2018b) but is congruent with an analysis of 353 nuclear markers (Monro et al., in preparation). That incongruence with Wu et al. (2013), (2015), (2018b) may be attributed to the insufficient phylogenetic information derived from limited gene loci used in their studies (Guo et al., 2022; Steenwyk et al., 2023). Short branches were consistently recovered for species within *Boehmeria* s.l., suggesting rapid diversification, which corresponds to and may be associated with the relatively diverse range of biomes that they occupy, from the moist broadleaf forests of Asia to African xeric biomes.

Our results demonstrated that plastome data can be a reliable tool for phylogenetic reconstruction, given that the resulting phylograms were highly resolved. A similar conclusion can be reached from previous Urticaceae phylogenies based on plastome data (Wang et al., 2020; Li et al., 2021; Ogoma et al., 2022; Wu et al., 2022).

### 4.2 Plastome structural variation provides further support for phylogenetic relationship

#### 4.2.1 Diverse plastome structural variation in *Boehmeria* s.l.

Considerable plastome size variation was detected for this group, which ranged from 142,627 bp to 170,958 bp, representing both the smallest and the largest ones recorded for the Urticaceae to date (NCBI, 2024). This is greater than the range of 145,419–161,930 bp of 57 plastomes in the Urticeae tribe (Ogoma et al., 2022) and is indicative of distinct structural differences (Ruhlman and Jansen, 2018). Changes in IR size are considered one of the main causes of plastome size variation (Jansen and Ruhlman, 2012), and in our study, they contribute to the large plastomes documented for clade B2. It has been suggested that large IR increases the stability of plastomes through homologous recombination-induced repair mechanisms (Maréchal and Brisson, 2010; Wicke et al., 2011; Zhu et al., 2016). However, we observed a lower GC content in this clade (B2). Lower GC content in larger plastomes has also been observed in *Pelargonium*, *Plantago*, and *Silene* (Magee et al., 2010; Zhu et al., 2016), where it has been ascribed to natural selection responding to the higher biochemical costs for GC base synthesis (Šmarda et al., 2014).

Gene loss and duplication was a distinct variation of clade C-B2, while clades B1 and B3-D were conserved in gene content. The duplications observed in clade B2 were mainly concentrated in the *rpl*, *rps*, and *psb* genes (**Figure 4**). These genes are involved in self-replication and photosynthesis, respectively (Zhang et al., 2020). The duplication of these functional genes may strengthen their adaptive ability to a wide range of habitats, from moist broadleaf forests to xeric deserts, by improving their stress response and light utilization capacity (Saha et al., 2017; Xiong et al., 2021). These gene losses and duplications were all caused by IR boundary shifts, which are common in plants (Dugas et al., 2015; Sinn et al., 2018; Guo et al., 2021; Ogoma et al., 2022; Wu et al., 2024). The loss of *trnL-UAG* in *B. tomentosa* was the only observed gene loss in clade B3-D. It occurred independent of any IR boundary shift, and since it is restricted to a single species, it has no phylogenetic implication. Compared with the divergent clade C-B2, clades B1 and B3-D had no obvious IR boundary shifts and had conserved IR size, boundary type, and gene content. The shift of IR/SC boundaries may, therefore, be linked to the diversification of clade C-B2. Considering the distinct features of gene loss and duplication and IR contraction and expansion among these clades, they could be used as effective phylogenetic informative characters.

Clade C-B2 also differed from its sister clades in the presence of a shared *trnC-GCA* inversion that may be associated with a key evolutionary event (Jansen and Palmer, 1987), which led to the divergence of clade C-B2 from B1. Our results recovered very rare large-scale inversions in Urticaceae occurring in the plastomes of *B. spicata*, *B. clidemioides*, and *B. umbrosa* (clade B2), which are however very common in other families such as Campanulaceae (Knox, 2014), Geraniaceae (Röschenbleck et al., 2017; Ruhlman and Jansen, 2018), Fabaceae (Charboneau et al., 2021; Lee et al., 2021), Passifloraceae (Rabah et al., 2019), and Juncaceae (Wu et al., 2024). Inversions have been demonstrated to represent homoplasies (Pinaceae, Wu et al., 2011; Fabaceae, Charboneau et al., 2021; Passifloraceae, Rabah et al., 2019) and homologies (Asteraceae, Jansen and Palmer, 1987; Geraniaceae, Röschenbleck et al., 2017; Poaceae, Wu et al., 2024; Campanulaceae, Xu et al., 2022). In this study, the *trnC-GCA* inversion was homologous for clade C-B2, and the large-scale inversion *trnH-GUG*_*trnK-UUU* was homologous for the clade comprising *B. clidemioides* and *B. umbrosa*. In contrast, the large-scale *trnH-GUG*_*ndhC* inversion and single *ndhF* inversion occurred independently in *B. spicata* and *C. macrocephalus*, respectively, which need deeper investigation with more species diversity.

#### 4.2.2 Trends of variations in the plastome evolution of *Boehmeria* s.l.

Plastomes of *Boehmeria* s.l. exhibit extensive variation in genome size, from gene content to GC content. Moreover, our results showed a general trend of size variation where the early clades (B1 and B3-D) were relatively conserved in evolution, whereas the later diverging groups (clades C and B2) showed a high degree of variation. This pattern was also observed for gene content, IR contraction and expansion, and IR boundary types and inversions. The plastome structural variation that we documented may be associated with its diversification and evolution that enabled members of this group to shift biome to more xeric environments and to exhibit diverse morphology and life forms (from herbs to trees). Diverse plastome structural variations were also detected in Poales, and these variations exhibit a trend of small–large–moderate pattern (Wu et al., 2024). Since the plastome bears the primary photosynthetic function, which is fundamental to the survival of green plants, knowing plastome evolution would be highly useful for us to better understand the evolutionary process of these plants.

## Data availability statement

The datasets presented in this study can be accessed at NCBI GenBank; the list of SRA accession numbers can be found in **Supplementary Table 1** and plastome accession numbers can be found in **Supplementary Table 2**.

## Author contributions

MZ: Formal analysis, Visualization, Writing – original draft, Writing – review & editing. LX: Data curation, Formal analysis, Writing – review & editing. J-JZ: Investigation, Resources, Writing – review & editing. QZ: Resources, Writing – review & editing. X-MQ: Methodology, Writing – review & editing. X-WL: Investigation, Writing – review & editing. LW: Conceptualization, Funding acquisition, Project administration, Resources, Supervision, Writing – review & editing. AM: Conceptualization, Writing – review & editing. L-FF: Conceptualization, Writing – review & editing.

## Supplementary material

Supplementary Figure 1 Phylogenetic relationships of *Boehmeria* and its related genera inferred by matrix2 (59-CDS): **(A)** concatenated tree produced by Maximum Likelihood (ML) analysis, numbers associated with branches (ML_BS/BI_PP) are assessed by Maximum Likelihood Bootstrap (ML_BS) and Bayesian posterior probabilities (BI_PP); **(B)** coalescent tree, numbers associated with branches denote local posterior probability (LPP) support values, pie charts show relative frequencies of the three quartet topologies around the branch (purple = congruent with the species tree, orange = first alternative topology, green = second alternative topology). Branches with no support values are maximally supported.

Supplementary Figure 2 Phylogenetic relationships of *Boehmeria* and its related genera inferred by matrix3 (gene+IGS): **(A)** concatenated tree produced by Maximum Likelihood (ML) analysis, numbers associated with branches (ML_BS/BI_PP) are assessed by Maximum Likelihood Bootstrap (ML_BS) and Bayesian posterior probabilities (BI_PP); **(B)** coalescent tree, numbers associated with branches denote local posterior probability (LPP) support values, pie charts show relative frequencies of the three quartet topologies around the branch (purple = congruent with the species tree, orange = first alternative topology, green = second alternative topology). Branches with no support values are maximally supported.

Supplementary Figure 3 Phylogenetic relationships of *Boehmeria* and its related genera inferred by matrix4 (gene+IGS-SNP): **(A)** concatenated tree produced by Maximum Likelihood (ML) analysis, numbers associated with branches (ML_BS/BI_PP) are assessed by Maximum Likelihood Bootstrap (ML_BS) and Bayesian posterior probabilities (BI_PP); **(B)** coalescent tree, numbers associated with branches denote local posterior probability (LPP) support values, pie charts show relative frequencies of the three quartet topologies around the branch (purple = congruent with the species tree, orange = first alternative topology, green = second alternative topology). Branches with no support values are maximally supported.

Supplementary Figure 4 Mauve alignment showing gene arrangements within the four clades with *Hemistylus odontophylla* (MN189963) used as reference (length indicated above). Large colored boxes represent the gene blocks and the colored lines indicate the linear position of different genes in the plastome.
