# Supplementary figures and images for: Polyphyly of Boehmeria (Urticaceae) congruent with plastome structural variation
Source: Front Plant Sci. 2024 Jul 30;15:1297499. doi: 10.3389/fpls.2024.1297499 (PMC11319286; doi:10.3389/fpls.2024.1297499)

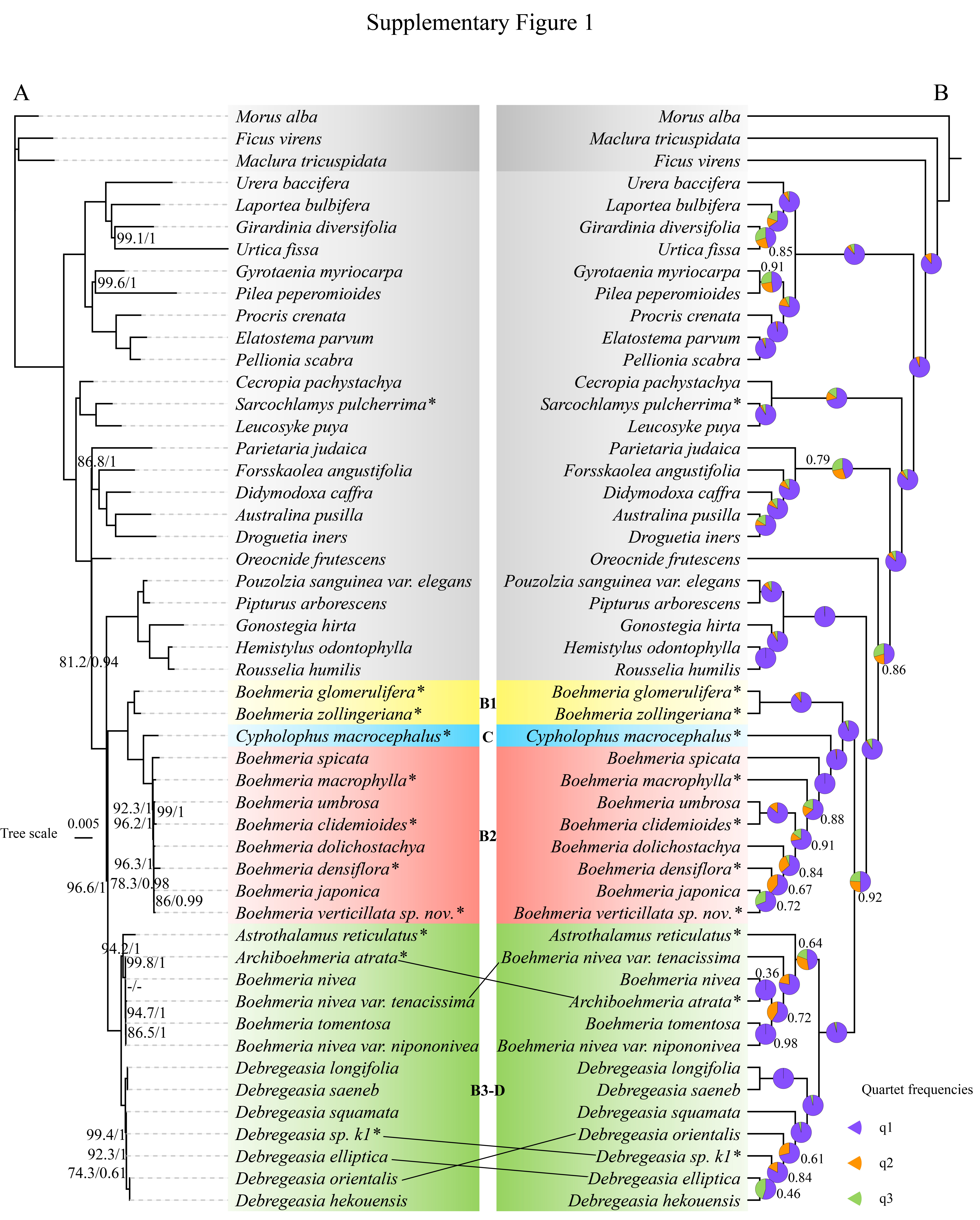

Supplement: Supplementary Figure 1 — Phylogenetic relationships of Boehmeria and its related genera inferred by matrix2 (59-CDS): (A) concatenated tree produced by Maximum Likelihood (ML) analysis, numbers associated with branches (ML_BS/BI_PP) are assessed by Maximum Likelihood Bootstrap (ML_BS) and Bayesian posterior probabilities (BI_PP); (B) coalescent tree, numbers associated with branches denote local posterior probability (LPP) support values, pie charts show relative frequencies of the three quartet topologies around the branch (purple = congruent with the species tree, orange = first alternative topology, green = second alternative topology). Branches with no support values are maximally supported. [file Image_1.jpeg]

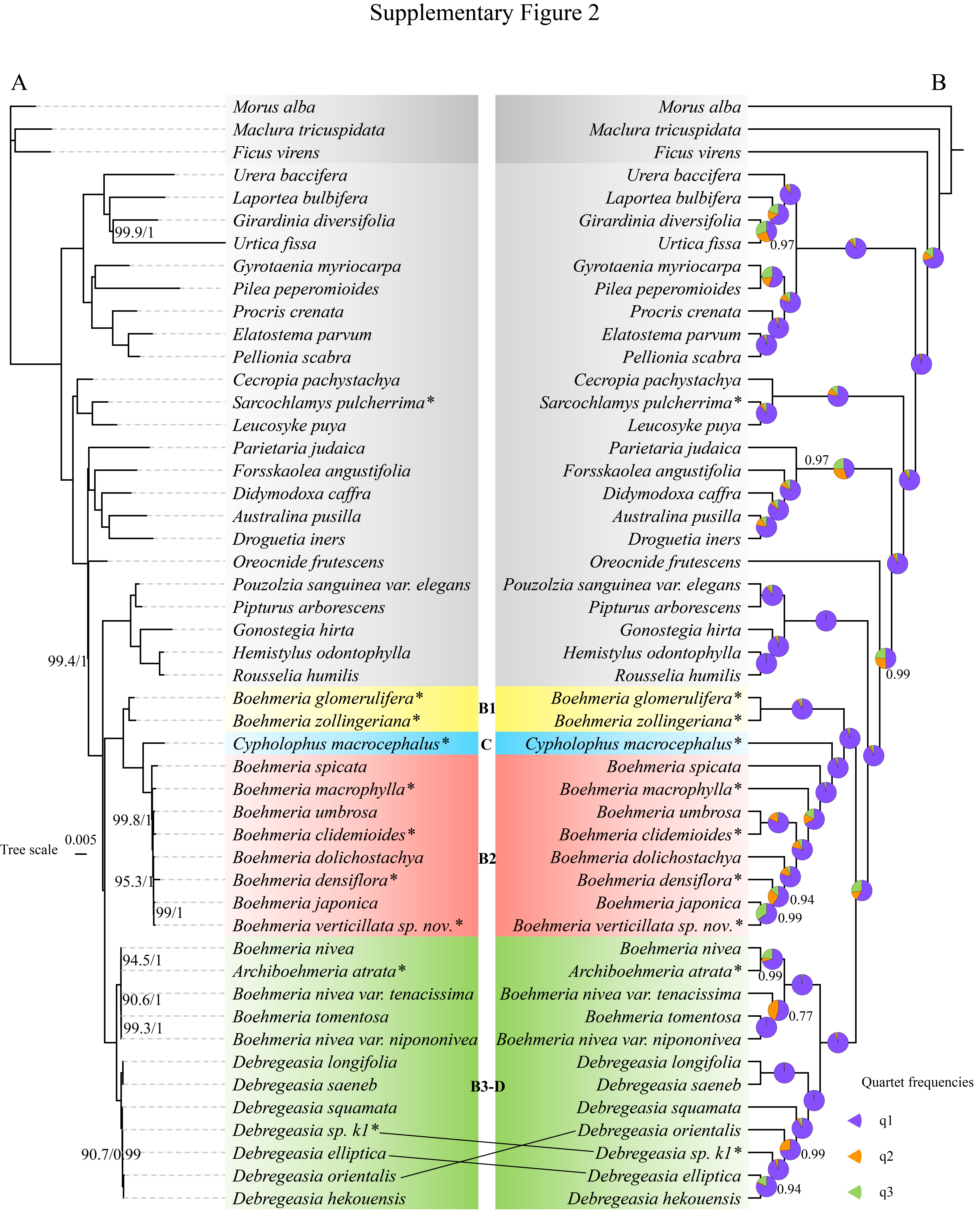

Supplement: Supplementary Figure 2 — Phylogenetic relationships of Boehmeria and its related genera inferred by matrix3 (gene+IGS): (A) concatenated tree produced by Maximum Likelihood (ML) analysis, numbers associated with branches (ML_BS/BI_PP) are assessed by Maximum Likelihood Bootstrap (ML_BS) and Bayesian posterior probabilities (BI_PP); (B) coalescent tree, numbers associated with branches denote local posterior probability (LPP) support values, pie charts show relative frequencies of the three quartet topologies around the branch (purple = congruent with the species tree, orange = first alternative topology, green = second alternative topology). Branches with no support values are maximally supported. [file Image_2.jpeg]

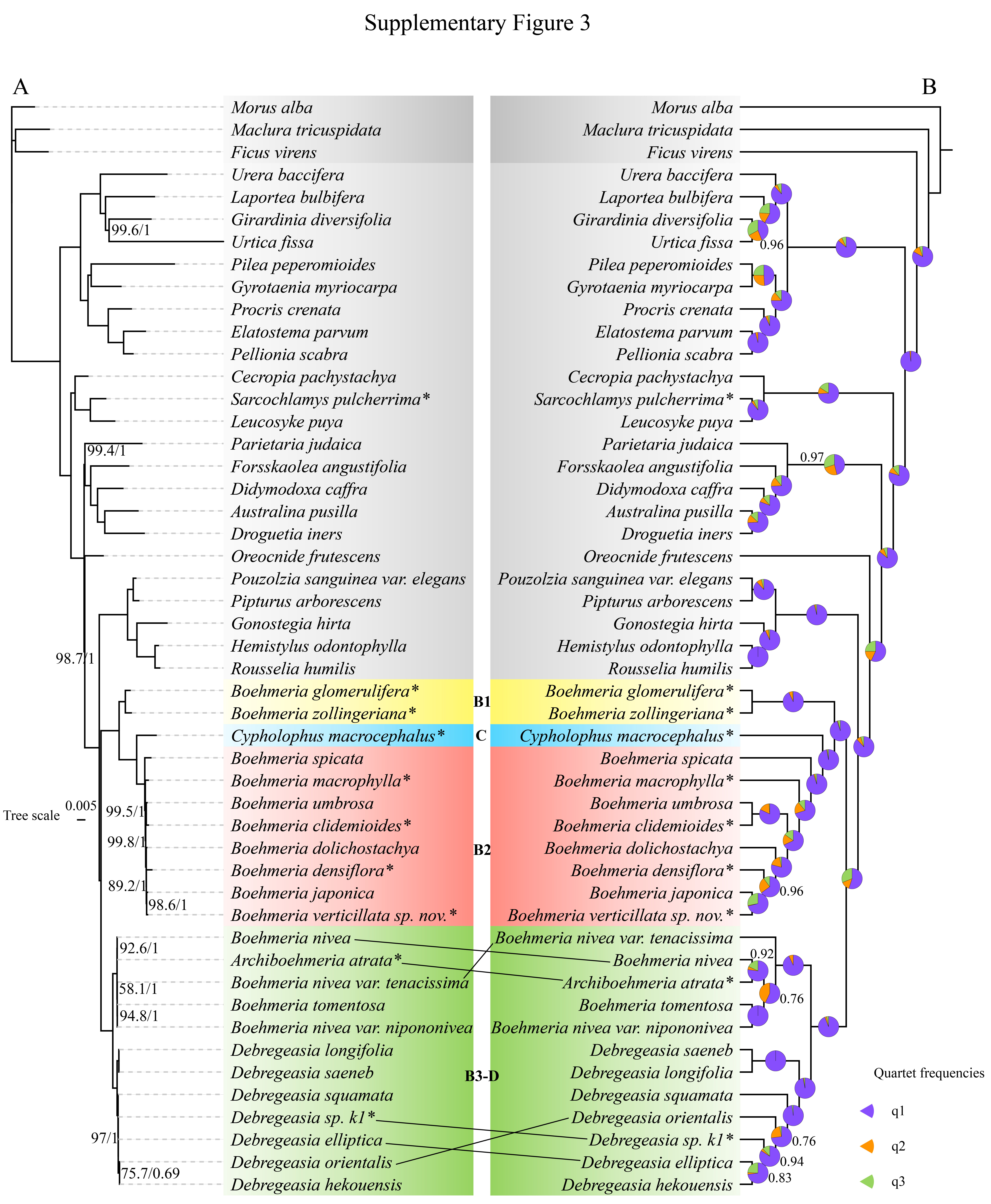

Supplement: Supplementary Figure 3 — Phylogenetic relationships of Boehmeria and its related genera inferred by matrix4 (gene+IGS-SNP): (A) concatenated tree produced by Maximum Likelihood (ML) analysis, numbers associated with branches (ML_BS/BI_PP) are assessed by Maximum Likelihood Bootstrap (ML_BS) and Bayesian posterior probabilities (BI_PP); (B) coalescent tree, numbers associated with branches denote local posterior probability (LPP) support values, pie charts show relative frequencies of the three quartet topologies around the branch (purple = congruent with the species tree, orange = first alternative topology, green = second alternative topology). Branches with no support values are maximally supported. [file Image_3.jpeg]

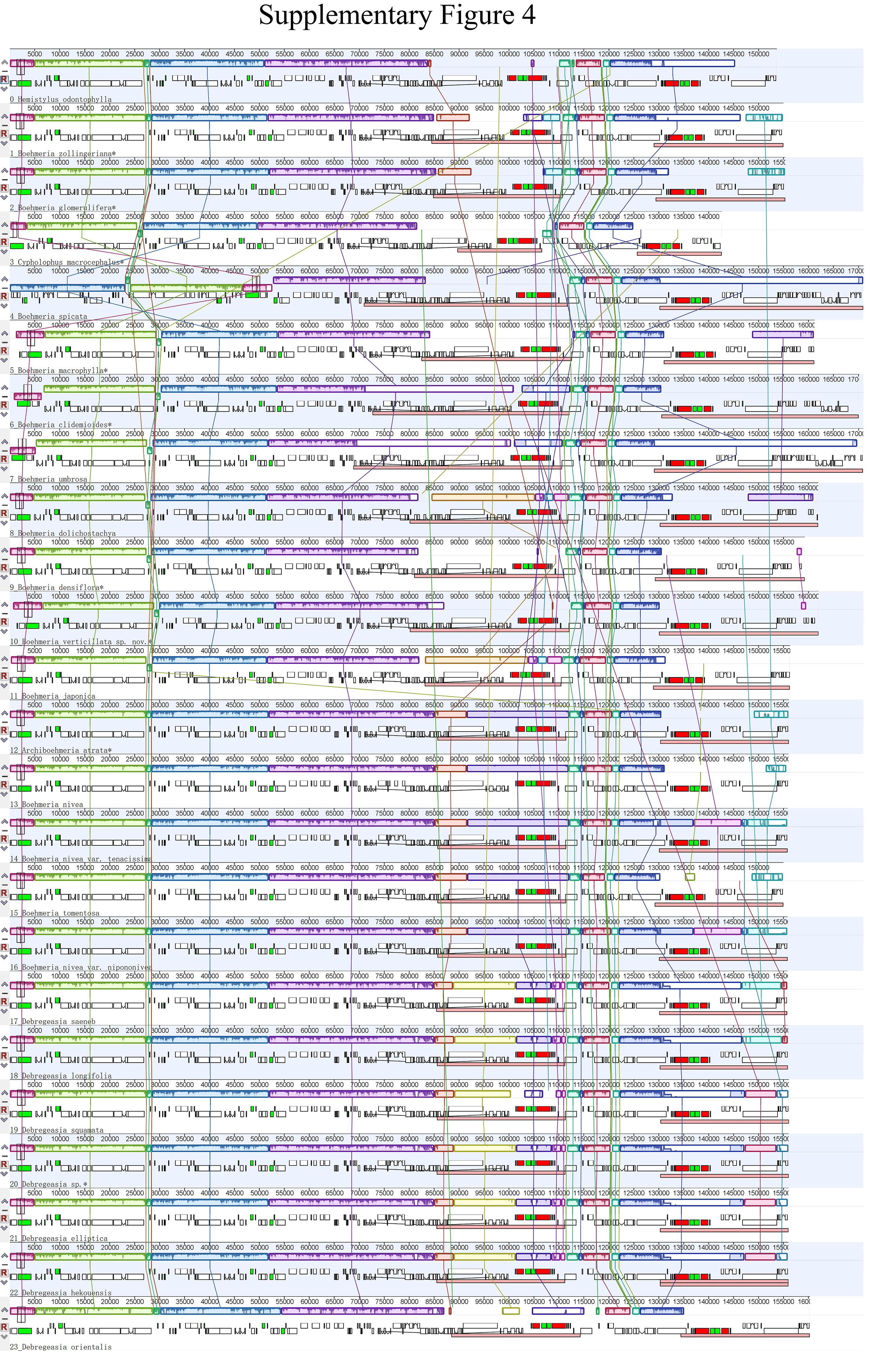

Supplement: Supplementary Figure 4 — Mauve alignment showing gene arrangements within the four clades with Hemistylus odontophylla (MN189963) used as reference (length indicated above). Large colored boxes represent the gene blocks and the colored lines indicate the linear position of different genes in the plastome. [file Image_4.jpeg]
